# Bacteremia caused by *Comamonas kerstersii* in a patient with acute perforated appendicitis and localized peritonitis: case report and literature review

**DOI:** 10.3389/fmed.2023.1283769

**Published:** 2023-12-07

**Authors:** Yingmiao Zhang, Kun Li, Yu Zhan, Lifeng Shi, Yi Zeng, Hui Wang, Zhongxin Lu

**Affiliations:** ^1^Department of Medical Laboratory, The Central Hospital of Wuhan, Tongji Medical College, Huazhong University of Science and Technology, Wuhan, China; ^2^Cancer Research Institute of Wuhan, The Central Hospital of Wuhan, Tongji Medical College, Huazhong University of Science and Technology, Wuhan, China

**Keywords:** *Comamonas kerstersii*, bacteremia, perforated appendicitis, 16S rRNA, case report

## Abstract

*Comamonas kerstersii* (*C. kerstersii*) is a Gram-negative bacterium that was initially thought to be non-pathogenic to humans and is abundant in the environment. In recent years, with the availability of matrix-assisted laser desorption ionization-time of flight mass spectrometry (MALDI-TOF MS) that enable fast and accurate bacterial identification, there have been increasing number of reports of human infections caused by *C. kerstersii*, indicating that this organism has emerged as human pathogen. In fact, most clinical isolates of *C. kerstersii* are recovered from peritoneal liquid, and bacteremia has been infrequently reported. Here, we report a case of bacteremia caused by *C. kerstersii* in a 28-year-old male patient with acute perforated appendicitis and localized peritonitis and present a comprehensive review of *C. kerstersii* infections in pathogenic diagnosis and clinical treatment as well as prognosis, thus providing a better understanding of *C. kerstersii*-related infections.

## Introduction

*Comamonas kerstersii* is an aerobic Gram-negative bacillus that belongs to the genus *Comamonas*. It was reclassified from *Comamonas terrigena* (*C. terrigena*) DNA group 3 and described as *C. kerstersii* in 2003 (1). Up to date, there have been 25 species with validly published and correct names under the List of Prokaryotic names with Standing in Nomenclature (LPSN) (2), most of which were recovered from the environment sources, such as soil, water, and plant (3). Among those *Comamonas* spp., only five species were involved in human infections, of which *Comamonas testosteroni* (*C. testosteroni*) remains the most, followed by *C. kerstersii* (3). Currently, with the development of mass spectrometry and 16S rRNA gene sequencing techniques, a growing number of bacterial species have been accurately classified, making the number of known species increase year by year. Therefore, reports of infectious cases caused by *C. kerstersii* have been gradually increased.

Since the first case of intra-abdominal infection duo to *C. kerstersii* was reported by Almuzara et al. in 2013 (4), there have been dozens of cases about *C. kerstersii*-related human infections (5–9). The majority of reported cases demonstrated the association of acute perforated appendicitis with polymicrobial infections, including *C. kerstersii*, *Escherichia coli*, and *Streptococcus* spp. In addition, Almuzara et al. successively reported the first case of urinary tract infection, psoas abscess, and pelvic peritonitis caused by *C. kerstersii* (10, 11). Nevertheless, *C. kerstersii* bacteremia has been infrequently reported. In this study, we report a case of bacteremia caused by *C. kerstersii* in a patient with acute perforated appendicitis and localized peritonitis and review the clinical characteristics of such infection of previously reported cases.

## Case presentation

A 28-year-old male patient came to our hospital with continuous abdominal pain and abdominal distension, but without fever, nausea, vomiting, or diarrhea. The abdominal pain was not relieved after administration of hydrotalcite chewable tablets. The patient was in good health with no medical conditions other than a history of mixed hemorrhoidal bleeding. On admission, the patient had tenderness in the right lower abdomen without rebound pain, Murphy’s sign was negative, and no other obvious abnormalities were observed. The patient had a temperature of 36.5°C, a pulse rate of 95 beats/min, and a blood pressure of 152/96 mmHg. Laboratory tests revealed the follows: White blood cell count of 20.67 × 10^9^/L (normal 3.5–9.5 × 10^9^/L), a neutrophil percentage of 91% (normal 40–75%), procalcitonin of 15.18 ng/mL (normal 0–0.046 ng/mL), apolipoprotein B of 1.59 g/L (normal 0.69–1.05 g/L), triglycerides of 3.42 mmol/L (normal 0–1.7 mmol/L), total cholesterol of 6.73 mmol/L (normal 0–5.18 mmol/L), low density lipoprotein (LDL) of 4.24 mmol/L (normal 0–3.37 mmol/L), free fatty acids (FFA) of 1.02 mmol/L (normal 0.17–0.58 mmol/L). The abdominal CT revealed a thickened and swollen appendix with fluid accumulation in the lumen, fecalith impaction with surrounding exudate. A comprehensive diagnosis of acute purulent appendicitis with perforation was made. The patient was empirically treated with intravenous levofloxacin hydrochloride sodium chloride solution (0.2 g/ 12 h). Prior to drug administration, one pairs of blood samples were drawn for microbiological test. After 2 days of incubation, the blood culture bottle was tested positive. The content from blood culture bottles were immediately inoculated onto Columbia blood agar, MacConkey agar, chocolate agar, and Sabouraud agar (Guangzhou Dijing Microbial Technology Co., Ltd., Guangzhou, China) at 35°C in presence of 5% CO_2_.

**Figure 1:**
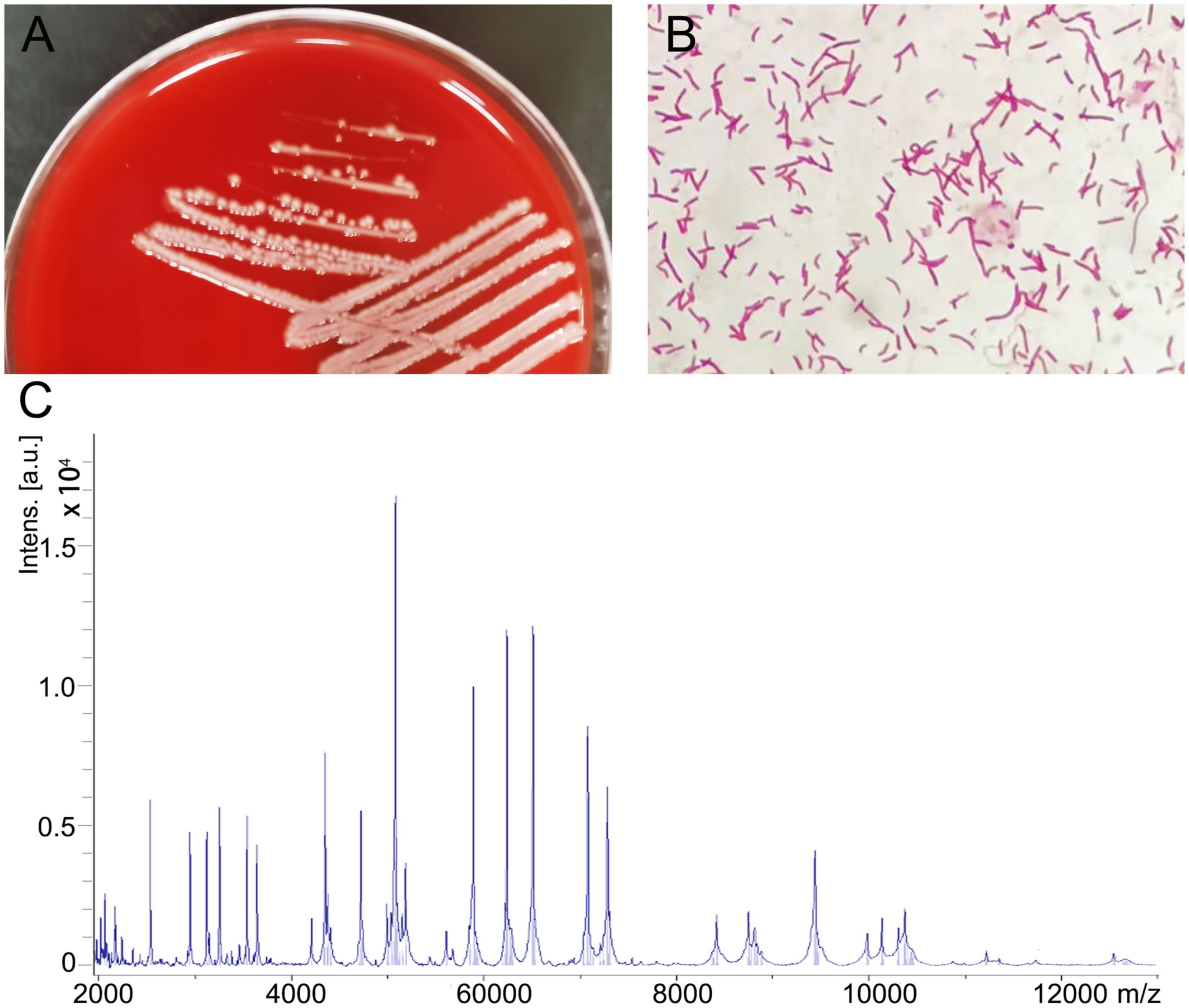
Isolation and identification of *Comamonas kerstersii* strain BC020423. **(A)** Bacterial colonies on Columbia blood agar after being cultured at 37°C in the presence of 5% CO_2_ for 24 h. **(B)** The gram-staining reveal that strain BC020423 is gram negative rods. **(C)** The spectrogram of the strain acquired by MALDI-TOF MS.

After 24 h of incubation, some round, moist, and white colonies grew on the Columbia blood agar and the single strain was named BC020423 (Figure 1A). Subsequently, the strain BC020423 was subjected to Gram staining and Gram-negative bacillus was observed under microscopy (Figure 1B). Then, the fresh colonies were selected and smeared on the microarray target and classified using MALDI-TOF MS (Bruker Daltonik GmbH, Germany) platform after a series of pretreatment according to the manufacturer’s instructions (Figure 1C). MALDI-TOF MS identified strain BC020423 as *C. kerstersii* with a high confidence score of 2.052.

**Figure 2:**
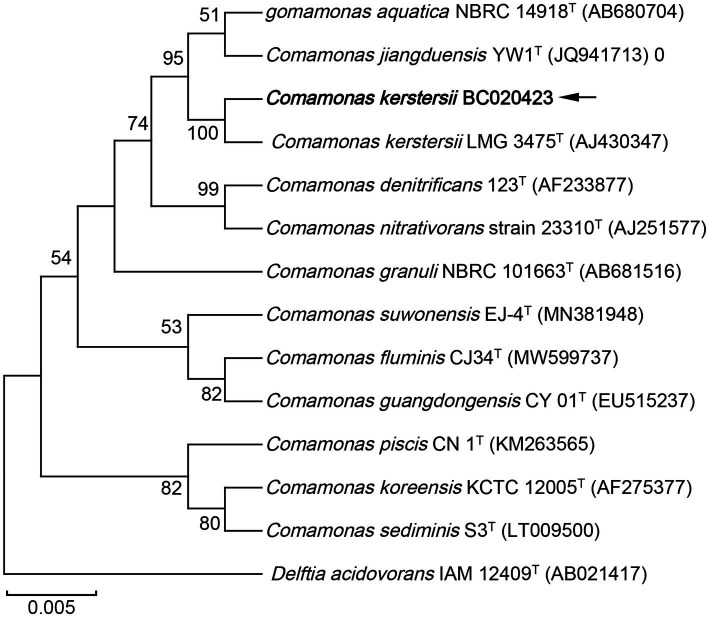
Phylogenetic tree based on the 16S rRNA gene sequences showing the relationship of isolated strain BC020423 (black arrow) and members within genus *Comamonas*. The tree was reconstructed by the neighbor-joining method, and *Delftia acidovorans* IAM 12409^T^ (AB021417) was used as an outgroup. Bootstrap values (>50%) based on 1,000 replicates are shown at branch nodes. T, type strain.

To further investigate the phylogenetic features of the strain BC020423 in this study, 16S rRNA gene sequencing was performed using universal primers (27F: 5’-AGTTTGATCMTGGCTCAG-3′, 1492R: 5’-GGTTACCTTGTTACGA CTT-3′). A total of 1,426 contiguous nucleotides were obtained. The complete 16S rRNA sequence of the strain BC020423 was analyzed with the EzBioCloud Database (12). The strain BC020423 exhibited the highest (99.40%) 16S rRNA gene sequence similarity with the type strain of *C. kerstersii* LMG 3475^T^ (GenBank accession no. AJ430347). The 16S rRNA sequencing results were submitted to GenBank (accession no. OR150448). Multiple alignments of the sequences from the related *Comamonas* and were carried out using MUSCLE algorithm (13). The phylogenetic tree was constructed on MEGA software using neighbor-joining (N-J) method (14). According to the phylogenetic tree, strain BC020423 was clustered with the type strain *C. kerstersii* LMG 3475^T^ with a bootstrap value of 100% (Figure 2). The results of the 16S rRNA gene sequencing indicated that the isolated strain BC020423 belongs to the *C. kerstersii* species.

Antimicrobial susceptibility test (AST) was performed using Vitek II automated system (bioMerieux), and the drug susceptibility was determined according to the Clinical and Laboratory Standards Institute 2022 (CLSI 2022) categories (15). The results showed that the strain BC020423 is sensitive to imipenem, meropenem, cefuroxime, ceftazidime, ceftriaxone, cefepime, piperacillin/tazobactam, and co-trimoxazole, and is resistant to ciprofloxacin, levofloxacin, and aztreonam. Besides, the susceptibility of this strain to gentamicin and amikacin remains at an intermediate level (Table 1). It is worth mentioning that the patient was empirically treated with intravenous levofloxacin (0.2 g/ 12 h), but the therapeutic effect was not favorable. Based on the AST result, the antimicrobial drug was changed to ceftriaxone (1.0 g/12 h). The patient underwent laparoscopic appendectomy with no postoperative complications on day 2 after his diagnosis. After 1 week of symptomatic and supportive treatment, the patient was discharged with normalized physiological conditions.

**Table 1:** Antimicrobial susceptibility of strain BC020423.

Antimicrobial agent	MICs (mg/L)	Category^a^
Amikacin	32	I
Aztreonam	≥ 32	R
Ciprofloxacin	≥ 4	R
Imipramine	≤ 0.25	S
Meropenem	≤ 0.25	S
Gentamicin	8	I
Cefuroxime	≤ 2	S
Ceftazidime	≤ 1	S
Ceftriaxone	≤ 1	S
Cefepime	≤ 1	S
Levofloxacin	≥ 8	R
Co-trimoxazole	≤ 2	S
Piperacillin-tazobactam	≤ 4	S

^a^S, sensitive; I, intermediate; R, resistant.

## Discussion

The acute appendicitis is one of the most common surgical acute abdomens worldwide, which can take place at any age. The annual incidence of appendicitis is approximately 233 per 100,000 population, and the lifetime incidence risk ranges from 6.7 to 8.6% (16). However, the pathogenesis underlying acute appendicitis still remains poorly understood. Bhangu et al. has summarized several causes of acute appendicitis, such as direct luminal obstruction, genetic effects, and environmental factors (17). The appendix is a critical organ that links to gastrointestinal tract, consisting of a large and diverse microbial community. There are a variety of pathogens that may cause appendicitis, including viral, bacterial, and fungal organisms (18). Among those pathogens, bacteria are considered to be major causative agents for the development of appendicitis. Reinisch et al. found that the most frequent organisms recovered from clinical samples of patients were *E. coli*, *Bacteroides* spp., and *Pseudomonas* spp. by summarizing the microbiological analysis of 584 patients with acute appendicitis (19). In addition, *Yersinia* spp. and *Campylobacter* spp. were also reported to be associated with appendicitis, but with rare cases (20, 21).

*Comamonas* spp. are causative agents of acute appendicitis with a widely geographic distribution, including Asia, Europe, Africa, and South America (3, 22). It is difficult to distinguish the members of genus *Comamonas* though phenotypic tests. The *C. kerstersii* is easily confused with *C. testosteroni* by manual or automatic bacterial identification systems currently available, such as Vitek 2 system and API 20NE system (4, 8), which may account for the absence of *C. kerstersii* case before. With the development of MALDI-TOF MS and 16S rRNA sequencing, more and more cases of *C. kerstersii* infection have been reported in recent years.

Since the first case of *C. kerstersii* related appendicitis reported in 2013, a total of 30 cases of *C. kerstersii* infection have been reported in different regions. We have reviewed all the cases and found that the type of specimen from these patients was predominantly peritoneal fluid or pus, accounting for 70% of all samples, while blood samples were reported in 7 cases (23.3%). Besides, one purulent material from psoas abscess and a urine sample from urinary tract infection were collected (10). The age of the 30 patients ranges from 5 to 84 years old, with a median age of 31.5 years. The prevalence ratio of *C. kerstersii* related appendicitis for male is 63.3%, which is higher than that of female. The country with the most reported cases is Argentina, followed by China. Among these cases, 12 patients were diagnosed with perforated appendicitis, and 4 patients were diagnosed with colon perforation. A single case of disease includes diverticulosis, psoas abscess, salpingitis, urinary tract infection, and cesarean section etc. It is worth noting that most cases are poly-microbial infections (73.3%) and the frequently isolated species are *Escherichia coli*, *Streptococcus* spp., and *Bacteroides fragilis*. In all cases, the diseases were controlled with appropriate surgery and/or antibiotic treatment and the patients had a good prognosis.

Bacteremia caused by *C. kerstersii* was rarely reported. Including the case in this study, there are seven cases of *C. kerstersii* bacteremia, of which only one case reported two bacterial species isolated from blood. The clinical characteristics and treatment options of the seven cases have been listed in Table 2. Fever and abdominal pain are the most common symptoms of acute appendicitis. Actually, a perforated appendix and localized inflammation can promote the entry of pathogenic bacteria into the bloodstream. Nevertheless, a number of cases with perforated appendix had not reported bacteremia, possibly duo to the absence of blood culture. The clinician roughly identifies the appendicular lesions through clinical symptoms and imaging examination, and then choose emergency surgery or abdominal drainage for treatment. The microbiological test of purulent materials from surgery or peritoneal liquid is able to identify pathogens in most cases, leading to a decrease in the use of blood cultures. One case reported a poly-microbial bacteremia by *C. kerstersii* and *Bacteroides fragilis* in a patient with diverticulosis who had ingested river water before his onset of symptoms, indicating that environmental material may serve as source of such pathogen (23). Rong et al. reported a case of bacteremia caused by *C. kerstersii* without identifiable origin of the organism (24). Although the patient had no abdominal symptoms or past infections that can be linked to this occurrence, the type 2 diabetes with diabetic neuropathy hinted his weakened immunity, which may contribute to the infection of *C. kerstersii*. A recent study reported maternal peripartum bacteremia caused by *C. kerstersii* following cesarean section, leading to rapidly progressing organ damage (25). These reports demonstrated the presence of *C. kerstersii* in the digestive tract and environment, highlighting the importance of identifying *C. kerstersii* in medical practice.

**Table 2:** Bacteremia related to *Comamonas kerstersii*.

Year	Gender	Age	Underlying disease	Clinical symptoms	Predisposing conditions	Surgery	Antibiotics	Country	Ref.
2014^a^	Male	65	Diabetes	Fever, chills, vomiting, diarrhea	Diverticulosis, ingested river water	No	CIP, IMP	Switzerland	(22)
2018	Male	31	No	Fever, abdominal pain, vomiting	Abdominal abscess, perforated appendix	Yes	CXM, MNZ	China	(6)
2019	Male	16	No	Fever, abdominal pain, nausea, vomiting	Appendicular gangrene and abscess	Yes	AMS, MNZ, TZP	Uruguay	(8)
2022	Male	8	No	Fever, right iliac fossa pain, nausea, vomiting	Abdominal abscess, perforated appendix	Yes	AMC, CN, MNZ	Morocco	(9)
2022	Male	82	Diabetes; colon adenoma	Fever, chills, constipation	No	No	TZP, CRO, AMC	Canada	(23)
2022	Female	29	No	High Fever, chills, abdominal pain	Cesarean section	No	MNZ, LEV, TZP, MEM	China	(2)
2023	Male	28	No	abdominal pain and distension	Localized peritonitis, perforated appendix	Yes	LEV, CRO	China	This case

^a^Polymicrobial bacteremia due to *C. kerstersii* and *Bacteroides fragilis*.Antibiotics: CIP, ciprofloxacin; IMP: Imipenem; CXM, cefuroxime; MNZ, metronidazole; AMS, ampicillin-sulbactam; TZP, piperacillin-tazobactam; AMC: amoxicillin-clavulanic acid; CN: gentamicin; CRO, ceftriaxone; LEV, levofloxacin; MEM, meropenem.

In our case, the patient was diagnosed with perforated appendicitis and localized peritonitis based on imaging examination and surgical exploration. *C. kerstersii* was gained from the blood culture of the patient prior to usage of antibiotics. The abdominal CT showed fecalith impaction inside the appendix, which is a common predisposing factor for acute appendicitis. Several indicators of blood lipid of the patient were elevated, which is related to his high blood pressure. Increased blood viscosity affects local microcirculation in the appendix and promotes inflammation. We speculate that *C. kerstersii* crosses the appendiceal wall and enters the bloodstream, thus triggering bacteremia, as well as causing tissue damage to the appendix and eventually causing localized peritonitis. The increased level of procalcitonin and leukocytosis indicate a severe inflammatory response in the body. The patient was empirically treated with levofloxacin, but the therapeutic effect was not favorable. The AST result showed that the isolate is resistant to fluorquinolone antibiotics and aztreonam, and remained at an intermediate level to aminoglycoside antibiotics. In fact, most clinical isolates of *Comamonas* spp. showed susceptible to a variety of antibiotics, including cephalosporins, carbapenems, and aminoglycosides antibiotics. *C. testosteroni* was reported to be resistance to ciprofloxacin, gentamicin, and ceftazidime in several cases (26–28). All of the patients were recovered after appropriate anti-infective therapy using metronidazole, piperacillin-tazobactam, and ceftriaxone etc. However, there were several cases of death that were associated with *C. testosteroni*, causing sepsis, purulent meningitis, and pneumonia (22, 29, 30).

## Conclusion

We describe a case of *C. kerstersii* bacteremia in a previously healthy male patient with acute perforated appendicitis and localized peritonitis and present a comprehensive review of *C. kerstersii*-related infections. The increasing cases of *Comamonas* infection highlights its potential of epidemic risk, which needs to be brought to the attention of clinicians. Timely detection of pathogens through peritoneal materials and blood and anti-infective therapy based on antibiotic susceptibility tests are critical for treatment of such infections. The techniques of MALDI-TOF MS and 16S rRNA gene sequencing may provide a fast and accurate identification of *Comamonas* spp. Moreover, the increase in the antibiotic resistance profile of *C. kerstersii* is a challenge for clinical treatment, thus further investigations are needed to elucidate the pathogenicity as well as epidemical characteristics of *C. kerstersii* infections.

## Data availability statement

The datasets presented in this study can be found in online repositories. The names of the repository/repositories and accession number(s) can be found at: https://www.ncbi.nlm.nih.gov/genbank/, OR150448.

## Ethics statement

The studies involving humans were approved by Medical Ethics Committee of the Central Hospital of Wuhan, Tongji Medical College, Huazhong University of Science and Technology. The studies were conducted in accordance with the local legislation and institutional requirements. The human samples used in this study were acquired from a by-product of routine care or industry. Written informed consent for participation was not required from the participants or the participants' legal guardians/next of kin in accordance with the national legislation and institutional requirements.

## Author contributions

YMZ: Conceptualization, Funding acquisition, Investigation, Writing – original draft. KL: Funding acquisition, Investigation, Writing – original draft. YZh: Formal analysis, Methodology, Writing – original draft. YZe: Formal analysis, Writing – original draft. LFS: Formal analysis, Investigation, Writing – original draft. HW: Conceptualization, Writing – original draft. ZXL: Conceptualization, Supervision, Writing – review & editing.

## References

[1] Wauters G De Baere T Willems A Falsen E Vaneechoutte M. Description of *Comamonas aquatica* comb. nov. and *Comamonas kerstersii* sp. nov. for two subgroups of Comamonas terrigena and emended description of *Comamonas terrigena*. Int J Syst Evol Microbiol. (2003) 53:859–62. doi: 10.1099/ijs.0.02450-0 12807213

[2] Parte AC Sardà Carbasse J Meier-Kolthoff JP Reimer LC Göker M. List of prokaryotic names with standing in nomenclature (LPSN) moves to the DSMZ. Int J Syst Evol Microbiol. (2020) 70:5607–12. doi: 10.1099/ijsem.0.004332, PMID: 32701423 PMC7723251

[3] Ryan MP Sevjahova L Gorman R White S. The emergence of the genus Comamonas as important opportunistic pathogens. Pathogens. (2022) 11:1032. doi: 10.3390/pathogens11091032, PMID: 36145464 PMC9504711

[4] Almuzara MN Cittadini R Vera Ocampo C Bakai R Traglia G Ramirez MS. Intra-abdominal infections due to *Comamonas kerstersii*. J Clin Microbiol. (2013) 51:1998–2000. doi: 10.1128/JCM.00659-13, PMID: 23576541 PMC3716083

[5] Biswas JS Fitchett J O'Hara G. Comamonas kerstersii and the perforated appendix. J Clin Microbiol. (2014) 52:3134. doi: 10.1128/JCM.00909-14, PMID: 24829228 PMC4136147

[6] Zhou YH Ma HX Dong ZY Shen MH. *Comamonas kerstersii* bacteremia in a patient with acute perforated appendicitis: a rare case report. Medicine (Baltimore). (2018) 97:e9296. doi: 10.1097/MD.0000000000009296, PMID: 29595695 PMC5895375

[7] Farfán-Cano G Parra-Vera H Ávila-Choez A Silva-Rojas G Farfán-Cano S. First identification in Ecuador of *Comamonas kerstersii* as an infectious agent. Rev Chilena Infectol. (2020) 37:179–81. doi: 10.4067/s0716-10182020000200179 32730486

[8] Palacio R Cabezas L Cornejo C Seija V. *Comamonas kerstersii* bacteremia in a young man with acute appendicitis. Revista chilena de infectologia: organo oficial de la Sociedad Chilena de Infectologia. (2020) 37:182–5. doi: 10.4067/s0716-10182020000200182, PMID: 32730487

[9] Bennani H El Ouarradi A Hanchi AL Soraa N. A young child with acute perforated appendicitis due to *Comamonas kerstersii*: a rare case report. Pan Afr Med J. (2022) 41:186. doi: 10.11604/pamj.2022.41.186.29615 35655686 PMC9120739

[10] Almuzara M Barberis C Veiga F Bakai R Cittadini R Vera Ocampo C. Unusual presentations of *Comamonas kerstersii* infection. New Microbes Infect. (2017) 19:91–5. doi: 10.1016/j.nmni.2017.07.003, PMID: 28794884 PMC5537401

[11] Almuzara M Cittadini R Estraviz ML Ellis A Vay C. First report of *Comamonas kerstersii* causing urinary tract infection. New Microbes Infect. (2018) 24:4–7. doi: 10.1016/j.nmni.2018.03.003, PMID: 29922468 PMC6004729

[12] Yoon SH Ha SM Kwon S Lim J Kim Y Seo H. Introducing EzBioCloud: a taxonomically united database of 16S rRNA gene sequences and whole-genome assemblies. Int J Syst Evol Microbiol. (2017) 67:1613–7. doi: 10.1099/ijsem.0.001755, PMID: 28005526 PMC5563544

[13] Edgar RC. MUSCLE: a multiple sequence alignment method with reduced time and space complexity. BMC Bioinfo. (2004) 5:113. doi: 10.1186/1471-2105-5-113, PMID: 15318951 PMC517706

[14] Tamura K Stecher G Kumar S. MEGA11: molecular evolutionary genetics analysis version 11. Mol Biol Evol. (2021) 38:3022–7. doi: 10.1093/molbev/msab120, PMID: 33892491 PMC8233496

[15] Clinical and Laboratory Standards Institute. Performance standards for antimicrobial susceptibility testing In: Clinical and Laboratory Standards Institute CLSI supplement M100. CLSI. 32nd ed. USA: Wayne (2022)

[16] Guan L Liu Z Pan G Zhang B Wu Y Gan T. The global, regional, and national burden of appendicitis in 204 countries and territories, 1990-2019: a systematic analysis from the global burden of disease study 2019. BMC Gastroenterol. (2023) 23:44. doi: 10.1186/s12876-023-02678-7, PMID: 36814190 PMC9945388

[17] Bhangu A Søreide K Di Saverio S Assarsson JH Drake FT. Acute appendicitis: modern understanding of pathogenesis, diagnosis, and management. Lancet (London, England). (2015) 386:1278–87. doi: 10.1016/S0140-6736(15)00275-5 26460662

[18] Lamps LW. Infectious causes of appendicitis. Infect Dis Clin North Am. (2010) 24:995–1018. doi: 10.1016/j.idc.2010.07.012 20937462

[19] Reinisch A Malkomes P Habbe N Bechstein WO Liese J. Bad bacteria in acute appendicitis: rare but relevant. Int J Colorectal Dis. (2017) 32:1303–11. doi: 10.1007/s00384-017-2862-0, PMID: 28710611

[20] Lamps LW Madhusudhan KT Greenson JK Pierce RH Massoll NA Chiles MC. The role of Yersinia enterocolitica and *Yersinia pseudotuberculosis* in granulomatous appendicitis: a histologic and molecular study. Am J Surg Pathol. (2001) 25:508–15. doi: 10.1097/00000478-200104000-00011, PMID: 11257626

[21] Campbell LK Havens JM Scott MA Lamps LW. Molecular detection of *Campylobacter jejuni* in archival cases of acute appendicitis. Modern pathology: an official journal of the United States and Canadian Academy of Pathology, Inc. (2006) 19:1042–6. doi: 10.1038/modpathol.3800640, PMID: 16715071

[22] Matsuda H Katayama I Watanabe T Komatsu A Iwakami N Goto Y. Vogesella urethralis-induced aspiration pneumonia and bacteremia in an elderly man: a first case report and literature review. BMC Infect Dis. (2023) 23:285. doi: 10.1186/s12879-023-08269-x, PMID: 37142952 PMC10157996

[23] Opota O Ney B Zanetti G Jaton K Greub G Prod'hom G. Bacteremia caused by *Comamonas kerstersii* in a patient with diverticulosis. J Clin Microbiol. (2014) 52:1009–12. doi: 10.1128/JCM.02942-13, PMID: 24371242 PMC3957752

[24] Rong K Delport J AlMutawa F. *Comamonas kerstersii* bacteremia of unknown origin. Case Rep Infect Dis. (2022) 2022:1–3. doi: 10.1155/2022/1129832 PMC8977328 35387092

[25] Qu H Zhao YH Zhu WM Liu L Zhu M. Maternal peripartum bacteremia caused by intrauterine infection with *Comamonas kerstersii*: a case report. World J Clin Cases. (2022) 10:7585–91. doi: 10.12998/wjcc.v10.i21.7585, PMID: 36158025 PMC9353907

[26] Reddy AK Murthy SI Jalali S Gopinathan U. Post-operative endophthalmitis due to an unusual pathogen, *Comamonas testosteroni*. J Med Microbiol. (2009) 58:374–5. doi: 10.1099/jmm.0.006072-0 19208890

[27] Bayhan G Tanır G Karaman I Ozkan S. *Comamonas testosteroni*: an unusual Bacteria associated with acute appendicitis. Balkan Med J. (2013) 30:447–8. doi: 10.5152/balkanmedj.2013.9135, PMID: 25207159 PMC4115943

[28] Hung YM Chang YT Kao CH. Polymicrobial bacteremia involving *Comamonas testosteroni* in a patient on Dialysis with acute appendicitis. Therapeutic apheresis and dialysis: official peer-reviewed journal of the International Society for Apheresis, the Japanese Society for Apheresis, the Japanese Society for Dialysis Therapy. (2017) 21:637–8. doi: 10.1111/1744-9987.12583, PMID: 29024403

[29] Swain B Rout S. *Comamonas testosteroni* bacteraemia in a tertiary care hospital. Indian J Med Microbiol. (2015) 33:602–3. doi: 10.4103/0255-0857.167325, PMID: 26470979

[30] Yasayancan N. The 20th *Comamonas Testosteroni* bacteremia case in the literature from Turkey: mortal and Polymicrobial a case report and literature review. Eurasian J Med Oncol. (2017) 1:168–171. doi: 10.14744/ejmo.2017.16878

